# Psychometric Properties of the Slovenian Version of Brief Sensation Seeking Scale

**DOI:** 10.3390/healthcare12010056

**Published:** 2023-12-26

**Authors:** Andrej Kastrin

**Affiliations:** Institute for Biostatistics and Medical Informatics, Faculty of Medicine, University of Ljubljana, SI-1000 Ljubljana, Slovenia; andrej.kastrin@mf.uni-lj.si

**Keywords:** sensation seeking, Brief Sensation Seeking Scale, BSSS-8, psychometric properties

## Abstract

Sensation seeking (SS) is a psychobiological personality trait characterized by an individual’s propensity to engage in various forms of risk-taking behavior. The Brief Sensation Seeking Scale (BSSS-8) is a widely used instrument for assessing SS that has been translated into several languages. However, only outdated and non-validated questionnaires have been used to measure SS in the Slovenian population. The aim of this study was to translate and psychometrically validate the Slovenian version of the BSSS-8. A total of 363 participants aged between 14 and 65 years completed the translated BSSS-8 and the questionnaire on drug abuse. The scale demonstrated good reliability (Cronbach’s α=0.81) and a unidimensional factorial structure as revealed by confirmatory factor analysis (CFA). The multigroup CFA showed gender-specific measurement invariance. In the nomological network, SS was positively associated with drug-related variables. The Slovenian version of the BSSS-8 scale is a short and simple instrument to assess SS for research and epidemiological purposes.

## 1. Introduction

Sensation seeking (SS from now on) has received considerable attention in the academic literature and has been recognized as one of the most studied personality traits in both healthy and psychiatric populations [1,2,3]. By formal definition, SS is a psychobiological personality trait that can be described as the “seeking of varied, novel, complex, and intense situations and experiences, and the willingness to take physical, social, and financial risks for the sake of such experience” ([2], p. 27). Individuals with high SS may seek more intense rewards, even if they are harmful or addictive, and are consequently inclined to engage in risky behaviors, such as gambling, experimenting with drugs, or speeding [4]. Therefore, reliable and valid measurements of SS are crucial for preventing unhealthy behavioral patterns.

Systematic research on SS was initiated in the mid-20th century through military experiments on sensory deprivation [3]. In the late 1960s, Zuckerman, a pioneer in SS, presented the first conceptual framework to describe SS behavior [1,2,3]. Zuckerman’s approach draws on the optimal level of arousal theory to explain individual differences in SS [1]. People have a preferred stimulus level, where some individuals seek intense arousal while others prefer low arousal. However, the research community did not fully support Zuckerman’s claims. Two decades later, he replaced the concept of optimal arousal with the optimal level of catecholaminergic activity in the limbic system [3]. This suggests that individuals have a specific level of catecholamine activity at which they are most comfortable and satisfied, and deviations from this level may drive their SS behavior. Empirical evidence indicates a high degree of trait heritability; however, the genetic background is not fully understood [5].

From a nomological perspective, SS is very similar to Eysenck’s psychoticism in terms of both impulsiveness and the propensity for new stimuli [6]. A review of the empirical results also shows associations with Extroversion, Openness to Experiences, and the dark triad traits (i.e., Machiavellianism, narcissism, and psychopathy) [7,8]. The literature has extensively examined the link between sensation seeking and risk behavior, including alcohol consumption [9], cigarette smoking [10], drug abuse [11], psychoactive substance use [12], risky sexual behavior [13], pathological gambling [14], extreme sports [15], aberrant driving behavior [16], and even delinquent behavior [17].

In psychometrics, SS is typically assessed using self-report questionnaires. Several instruments have been developed to measure this trait, either with complex global scales or specific questionnaires. For example, the Zuckerman–Kuhlman–Aluja Personality Questionnaire (ZKA-PQ) embeds a separate SS factor into a 200-item personality inventory [18]. On the other hand, Arnett [19] combined two dimensions, intensity and novelty, into a unified 20-item questionnaire, the Arnett Inventory of Sensation Seeking (AISS), to measure the construct of SS. The ZKA-PQ is too comprehensive for use in SS assessments, whereas the AISS has been criticized for its unacceptable reliability [20]. However, the Zuckerman’s Sensation Seeking Scale, Form V (SSS-V), has become a widely accepted standard for measuring sensation seeking [21]. The scale includes 40 forced-choice items and consistently reveals a four-dimensional structure [2]: Thrill and Adventure Seeking (TAS), Experience Seeking (ES), Disinhibition (Dis), and Boredom Susceptibility (BS). Example items are “I would like to try parachute jumping” (TAS) and “I can’t stand watching a movie that I’ve seen before” (BS). Despite its widespread use, the SSS-V has faced criticism. Researchers have complained about the length, colloquial language (i.e., many items are defined using an obsolete language [19,22]), and ipsative scoring (i.e., the forced-choice questionnaire format violates the assumption of item independence and makes the data unsuitable for standard factor analysis [23]) of the instrument. In addition, modern psychometry promotes the use of “super-short” personality measures, which are particularly useful in research settings with large sample sizes, online surveys, and studies investigating multiple personality constructs [24]. By using short questionnaire forms, we can overcome the problem of low response rates [25] or time constraints [26].

Several other scales have been developed to overcome the shortcomings of the SSS-V. Based on a subset of 10 items from Zuckerman’s SSS-IV questionnaire, Madsen et al. [27] made the first attempt and developed the Short Sensation-Seeking Scale. Psychometric validation of the scale was promising (i.e., high retest reliability and significant correlations with drug- and gender-related behaviors), but the research community did not adopt it. Hoyle et al. [28] later developed the Brief Sensation-Seeking Scale (BSSS-8) based on a subset of eight items from the SSS-V. The use of Likert-type items allowed the authors to solve issues related to ipsative scoring. The scale exhibited excellent item characteristics and high reliability across gender, age, and ethnicity. The BSSS-8 score has been shown to be a reliable predictor of several risky activities, including excessive alcohol consumption, smoking, and illicit drug use [9,28,29,30]. The BSSS-8 retains Zuckerman’s conceptualization of SS, structured around four dimensions with two items per dimension. The BSSS-8 has received substantial recognition from the research community. At the time of this writing, Hoyle et al.’s [28] work has been cited more than 1500 times.

The findings of studies that have assessed the metric properties of the BSSS-8 have been ambiguous regarding factor structure, with no agreement on the number of factors. Stephenson et al. [30] found a second-order unidimensional factor solution for English-speaking Latinos and a four-factor structure for Spanish-speaking Latinos. Chen et al. [31] adapted the BSSS-8 for Chinese speakers and found a four-factor structure. The high correlations between sensation seeking and various risk-taking behaviors provided evidence of concurrent validity. Primi et al. [32] adapted and validated the Italian version of the BSSS-8 for high school students. The authors confirmed a single-factor latent structure that was invariant across demographic groups, including age and gender. Criterion validity was confirmed to be significantly related to gambling behavior. Next, Ref. [33] adapted and validated the BSSS-8 with a large group of Spanish-speaking Peruvian adolescents. The authors demonstrated a unidimensional latent structure, and concurrent validity was assessed using alcohol, tobacco, and marijuana use. Pechorro et al. [34] conducted a validation study among adolescents at risk for criminal behavior in Portugal. The authors demonstrated that the single-factor structure of the BSSS-8 is an appropriate solution. They found associations between criminal tendencies and the dark triad traits. Martín-Fernández et al. [35] also showed the unidimensional structure of the BSSS-8 in young Spanish adults. Merino-Soto et al. [36] recently demonstrated similar findings. In summary, these studies highlight the need for further research on the dimensionality of the BSSS-8.

To date, only non-validated versions of the SSS-IV and SSS-V scales have been used to measure SS in the Slovenian population (e.g., [37]). In Slovenia, SS has not been systematically studied, and no instruments have been adapted or developed to measure the SS concept. The main goal of this study is to develop a Slovenian version of the BSSS-8 scale. Specifically, three objectives are pursued: (i) to translate the original English version of the BSSS-8 scale into Slovenian; (ii) to examine its metric properties, including factor structure, reliability, and metric invariance; and (iii) to examine the construct validity of the BSSS-8 scale through a nomological network, incorporating other personality and temperament variables.

## 2. Methods

**Figure 1:**
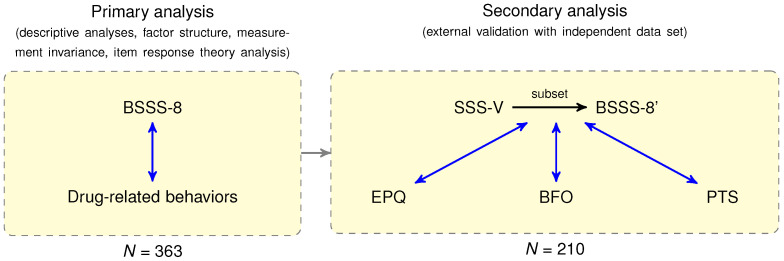
General workflow of study design. The main psychometric analysis of the Slovenian Brief Sensation Seeking Scale (BSSS-8) was based on a total of N=363 participants. The construct validity of the BSSS-8 was explored using a correlation analysis (blue lines) with drug-related behaviors. External validation, in terms of construct validity, was examined using an independent dataset composed of scores from four questionnaires (N=210): the Sensation Seeking Scale-Form V (SSS-V), Eysenck Personality Questionnaire (EPQ), Big Five Observer (BFO), and Pavlovian Temperament Survey (PTS) [38]. First, the corresponding BSSS-8 items were extracted from the collected SSS-V data, and the total score (BSSS-8′) was computed. Second, a correlation analysis was performed between the derived BSSS-8′ score and EPQ, BFO, and PTS measures. Finally, we created a nomological network and interpreted the construct validity.

The primary psychometric analysis of the Slovenian Brief Sensation Seeking Scale (BSSS-8), including descriptive analyses, factor structure, measurement invariance, was based on a total of 363 participants. The construct validity of the BSSS-8 was examined using correlation analysis with drug-related behaviors. Additionally, construct validity was examined using an independent dataset from our previous study [38]. The workflow of the study design is shown in Figure 1.

### 2.1. Participants

This study was conducted between October 2022 and January 2023. We recruited 377 participants of Slovenian nationality and Caucasian ethnicity. The majority of participants were recruited from three public secondary schools in Ljubljana, Slovenia. To increase the sample size, we asked participants to invite their family members. A total of 14 participants did not provide complete demographic data; therefore, they were removed from the dataset, resulting in a sample of 363 participants. The main demographic characteristics of the study participants are presented in Table 1. Owing to the highly imbalanced distribution of age categories, we also reported age as a binarized variable and used it in all the subsequent analyses.

Participation in the study was voluntary and anonymous. Participants were informed of the aims of the study and signed an informed consent form. Written parental consent was obtained from all participants aged less than 18 years. The detailed study protocol and methodology were approved by the National Medical Ethics Committee (protocol code 120-576/2020/3), and the study was conducted in strict accordance with the guidelines of the Declaration of Helsinki.

### 2.2. Materials

In this section, we first present the Brief Sensation Seeking Scale (BSSS-8) and the questionnaire used to assess participants’ experiences with drugs. Next, we describe the four instruments employed in our previous study and repurposed in this study to estimate the construct validity of the BSSS-8: Zuckerman’s Sensation Seeking Scale-Form V (SSS-V), the Eysenck Personality Questionnaire (EPQ), the Big Five Observer (BFO), and the Strelau Temperament Inventory (STI).

#### 2.2.1. Brief Sensation Seeking Scale

SS was measured using the Slovenian-translated version of the BSSS-8. The scale comprises two items for each of the following four factors: Thrill and Adventure Seeking (TAS), Experience Seeking (ES), Disinhibition (Dis), and Boredom Susceptibility (BS). Examples of items include “I enjoy doing scary things” (TAS) and “I like going to wild parties” (Dis). Responses were recorded on a five-point Likert scale ranging from 1 (*strongly disagree*) to 5 (*strongly agree*) such that the sum of all items ranged from 5 to 40. A higher total score indicates a higher level of SS. The original Hoyle’s scale [28] demonstrated acceptable reliability (Cronbach’s α=0.79). The validity of the scale was established by a positive correlation between the BSSS-8 score and drug abuse behavior [28]. The Slovenian translation of the questionnaire is provided in Appendix A Table A1.

#### 2.2.2. Drug-Related Behaviors

Participants responded to a series of six questions regarding their experiences with alcohol, tobacco, and illegal drugs. Similar questions were used in the original study by Hoyle et al. [28]. Participants first indicated whether they had consumed alcohol (question $A_{1}$) or smoked tobacco ($S_{1}$) and, if they had, the frequency of their use. The number of days of drinking in the last month was assessed using the question “Please type the number of days between 0 and 30 that you used alcohol in the last 30 days” ($A_{2}$). The likelihood to consume alcohol was assessed by asking ($A_{3}$) “How likely is it that you will drink alcohol in the next six months?” Similarly, the number of smoking days in the last month was assessed with the question ($S_{2}$) “Please type the number of days between 0 and 30 that you smoked tobacco in the last 30 days”. The propensity to smoke was determined with the question ($S_{3}$) “How likely is it that you will smoke in the next six months?” Finally, for the abuse of illegal drugs, we used two questions: “Please type the number of days between 0 and 30 that you used illegal drugs in the last 30 days” ($D_{1}$) and “How likely is it that you will use illegal drugs in the next six months?” ($D_{2}$).

Basic demographic information, including gender, age, and educational level, was also collected. Age was represented as a categorical variable with four categories (≤20, 21–40, 41–60, and >60 years).

#### 2.2.3. Sensation Seeking Scale-Form V

The Sensation Seeking Scale-Form V (SSS-V) consists of 40 forced-choice items, each with two options, from which the participant must select the one that best describes her or him [2]. As mentioned in the introduction, Zuckerman [2] proposed a multifactorial structure of sensation seeking reflected in four subscales: TAS, ES, Dis, and BS. Example items include “I like ‘wild’ uninhibited parties” (Dis) and “The worst social sin is to be a bore” (BS). The total score was calculated as the sum of the scores on all four subscales. Cronbach’s α coefficients for the original English version ranged from 0.72 to 0.91 [22,39]. In our validation sample (N=210), the α values ranged from 0.48 to 0.82 [38].

#### 2.2.4. Eysenck Personality Questionnaire-Revised Short Form

According to Eysenck’s PEN model, the three “supertraits” essential for explaining individual personality differences are Psychoticism (P), Extroversion (E), and Neuroticism (N) [40]. The Eysenck Personality Questionnaire (EPQ) contains subscales for all three dimensions as well as a Lie scale [41]. This study used the short version of the self-report questionnaire (EPQR-S), which consists of 48 items. Examples of items include “Are you a talkative person?” (E) and “Do you enjoy co-operating with others?” (P). Each item was scored on a binary scale, with a maximum score of 12 for each subscale. The internal consistency of the Slovenian standardization sample (unpublished) ranged from 0.63 to 0.86. In our validation sample, α ranged from 0.45 to 0.85 [38]. The validity of the questionnaire has been confirmed by numerous studies (e.g., [42]).

#### 2.2.5. Big Five Observer

We used the Big Five Observer (BFO), a self-report scale composed of 40 bipolar pairs of adjectives reflecting personality traits according to the Big Five model, including Openness, Conscientiousness, Energy, Agreeableness, and Neuroticism [43]. The adjectives were rated on a seven-point scale ranging from 1 (*not agree at all*) to 7 (*absolutely agree*). For example, the adjective pair “Shy–Brave” belongs to the energy scale; the closer the answer is to seven, the more brave the participant is. In our validation study, the α coefficients varied from 0.67 to 0.80 [38]. The validity of the Slovenian translation of the BFO was confirmed using a multitrait-multimethod approach [44]. The translated version showed acceptable reliability [45] and the α coefficients based on the Slovenian standardization sample (N=982) ranged from 0.67 to 0.85.

#### 2.2.6. Pavlovian Temperament Survey

The Pavlovian Temperament Survey (PTS) measures the topology of the nervous system as proposed by Pavlov [46,47]. PST is composed of three dimensions: Strength of Excitation (SE), Strength of Inhibition (SI), and Mobility (MO). SE is the ability of the central nervous system (CNS) to endure excessive stimulation without developing transmarginal inhibition. In contrast, SI refers to the ability of the CNS to tolerate a conditioned inhibition state. Finally, MO refers to the ability of the CNS to respond quickly to environmental changes. Example items include “I like very demanding jobs” (SE) and “I can hide my anger if needed” (SI). We administered the Slovenian adaptation of the PTS, which consists of 60 items [48]. Responses were provided on a five-point scale ranging from 1 (*strongly disagree*) to 5 (*strongly agree*). The α coefficients of the original Polish instrument were in the range 0.68–0.91 [46]. In our validation sample, α coefficients varied between 0.80 and 0.85 [38].

### 2.3. Procedure

For the linguistic translation of the BSSS-8, we used the standardized protocol for back-translation [49]. First, we translated the questionnaire from English into Slovenian. The translation was performed by the author. The native English speaker then provided a back-translation into English. The English translator was blinded to the context and purpose of the translated questionnaire. We checked all discrepancies between the original version and the Slovenian translation until no semantic differences were found. The data were collected using an anonymous online questionnaire. The entire testing session lasted approximately five minutes. We did not offer any material or financial incentives to the participants.

### 2.4. Data Analysis

All statistical computations were performed using the R software (v. 4.2.1) [50]. Only the cases with complete data were included in the analysis. We reported descriptive statistics and examined the mean differences in gender, age, and educational groups. We estimated reliability using Cronbach’s α and McDonald’s ω coefficients [51].

We tested our dataset for multivariate normality using the Henze–Zirkler test [52]. Exploratory factor analysis (EFA) was performed to examine the latent structure. The suitability of the data for conducting EFA was assessed using the Kaiser–Meyer–Olkin index (KMO) [53] and Bartlett’s test of sphericity [54]. We performed a parallel analysis using the nFactors package to estimate the most plausible number of latent factors underlying the set of observed variables [55]. For the EFA, we used the function fa() with default parameters from the psych package.

We used the lavaan package [56] to perform a confirmatory factor analysis (CFA). To assess the fit of the CFA models, we used the $\chi^{2}$-statistic, root mean square error of approximation (RMSEA), comparative fit index (CFI), and Tucker–Lewis index (TLI). The criteria for good model fit were a non-significant $\chi^{2}$ value, RMSEA < 0.06, CFI < 0.96, and TLI < 0.95. We considered the model acceptable when RMSEA < 0.08, CFI < 0.90, and TLI < 0.90 [57].

Next, we performed a multi-group CFA (MG-CFA) to determine the measurement equivalence in terms of gender and age groups. In this study, we adopted Brown’s procedure [58] to test measurement invariance. Measurement invariance typically comprises a series of model comparisons. Different levels of measurement invariance are described in the literature and should be tested sequentially, one after the other. First, configural invariance is the weakest mode of measurement invariance. This type of invariance examines the equivalence of factorial structures (i.e., it assumes that the factors have the same dimensionality and that the items can be assigned to the factors in all groups in the same manner). Configural invariance is a necessary condition for unbiased comparison between groups. More conservative modes of measurement invariance refer to (i) factor loadings (i.e., weak invariance) and (ii) intercepts of the items (i.e., strong invariance). Assuming weak invariance, the structural relationships between factors can be compared between groups (e.g., correlations). If we confirm strong invariance, we can compare the differences in the mean values of the factors between the groups. Finally, we tested for strict invariance to assess whether the residual variances of the items were the same between groups. In each subsequent step, we tested the hypotheses on group differences by including different restriction patterns for loadings and intercepts [59]. $\Delta \chi^{2}$ is typically used to assess the fit of two nested models. As with $\chi^{2}$, $\Delta \chi^{2}$ is very sensitive to sample size; therefore, we used it for descriptive purposes only, and based our conclusions on the ΔCFI criterion. ΔCFI>0.01 between successive models in the invariance test indicates a significant deterioration in model adequacy [60]. However, because of the different group sizes, the results of the ordinary MG-CFA bias the resulting parameter estimates towards a larger sample. Therefore, we report the results of the Monte Carlo procedure proposed by Yoon and Lai [61].

As part of the validity analysis, we used a correlation analysis to investigate the relationships between the total BSSS-8 score, drug-related variables, and demographic data. We visualized the correlations between variables using the qgraph package [62]. We also investigated the inter-individual variability of participants in the variables studied using t-distributed stochastic neighbor embedding (t-SNE), a dimensionality reduction method that maps a multivariate dataset into a low-dimensional vector space. The reader is referred to the paper by van der Maaten and Hinton [63] for further details.

For the item response theory (IRT) analysis, we used the graded response model (GRM) provided in the mirt package [64] to compute the item characteristics. For the GRM, we analyzed two parameters: discrimination and difficulty. Discrimination refers to the potential of an item to differentiate between participants at different trait levels, whereas difficulty refers to the amount of trait necessary to have a 50% chance of endorsing an item. A crucial feature of IRT modeling is the information that indicates the accuracy of the measurement along the trait continuum.

## 3. Results

The results section is divided into five parts. First, we present the basic descriptive statistics. Next, we perform an EFA to identify the underlying factor structure of BSSS-8. We then examine the generalizability of the factor model using CFA and explore the results of the MG-CFA to determine the measurement invariance of the BSSS-8. We continue with an investigation of the construct validity of the BSSS-8 through a nomological network and conclude the section with an IRT analysis.

### 3.1. Descriptive Analyses

First, we checked the data for skewness, kurtosis, possible outliers, and missing data. We considered skewness and kurtosis between 2.0 and 4.0 acceptable to demonstrate a univariate normal distribution. Fourteen cases with missing values were excluded from the dataset. Therefore, the dataset was reduced to 363 cases (297 females and 66 males). Table 2 presents the basic descriptive statistics for the BSSS-8 items. The mean score of the items was 2.80, with a *SD* of 1.28, indicating the tendency of participants to choose intermediate categories of items. All the items had low skewness [−0.24,−0.40] and kurtosis [−1.41,−0.70]. The mean correlation between the items and the total score was 0.60, indicating that the items were closely related to SS. The scale showed reasonable internal consistency (α=0.81; 95% CI [0.78,0.84]). We found no significant change in Cronbach’s α values when the individual items were removed. We also computed the ω coefficient (ω=0.81, 95% CI [0.79,0.84]) based on the polychoric correlation matrix.

Table 3 summarizes the descriptive statistics for each item of the BSSS-8, categorized by gender, age, and education groups. Table includes only the *p*-values corresponding to the *t*-test or *F*-test. Table A2 provides a detailed summary of the test statistics and effect size measures. The effect sizes are reported as Cohen’s *d* for the *t*-test and $\eta^{2}$ for the *F*-test. Cohen’s *d* is based on benchmarks for small (0.2), medium (0.5), and large (0.8) effects [65]. Cohen [65] suggested cutoff values for classifying $\eta^{2}$ as small (0.01), medium (0.06), and large (0.14).

Males scored significantly higher than females on items 1, 2, 3, and 7, although the effect sizes were small. A comparison between age groups showed that the differences were more pronounced. With the exception of items 2 and 8, all items showed significant differences between groups in favor of a group of young participants with small-to-large effect sizes. When comparing differences between educational levels, all items except item 2 showed significant differences between groups in favor of the group of young participants. Effect sizes ranged from small to large.

### 3.2. Factor Structure

The KMO index was meritorious (KMO=0.84), and Bartlett’s test indicated that the correlation matrix was relevant for the factor analysis ($\chi^{2}\left(28\right)=878.18$, p<0.001). The Henze–Zirkler test rejected multivariate normality as a plausible distribution for the dataset (HZ=1.41, p<0.001). Because of the non-normal data, we used a robust maximum likelihood estimator for both EFA and CFA. First, we used an EFA to examine the structure of BSSS-8. Parallel analysis (we used the 95th percentile as the basis for the comparison baseline and set the number of random datasets to 1000) revealed a single latent factor underlying the BSSS-8 items. Pairwise correlations among the BSSS-8 items ranged from 0.15 to 0.72, with the first eigenvalue ($\lambda_{1}=3.56$) dominating, providing further evidence for the single-factor model (see the last column in Table 2). High factor loadings (i.e., all loadings above 0.45) indicated a relatively high percentage of shared variance between the items and latent factor. The one-factor model explained 42% of the total item variance in SS.

**Figure 2:**
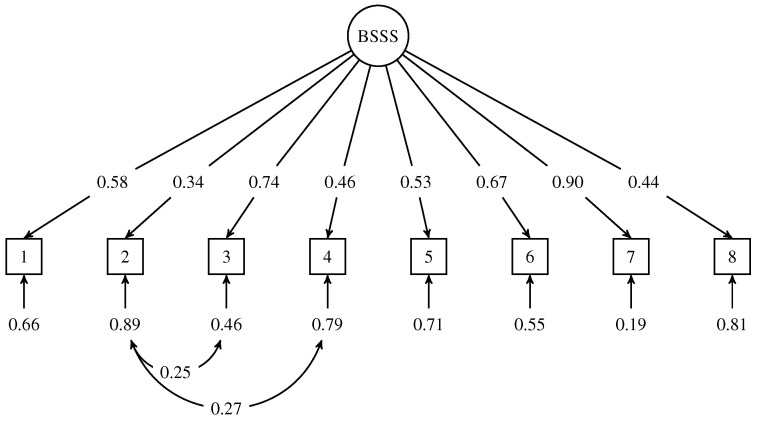
Optimized measurement model of Brief Sensation Seeking Scale (BSSS-8). The path diagram represents the standardized parameter estimates resulting from the confirmatory factor analysis applied to the BSSS-8 scale. The eight items, shown as boxes, measure the latent construct of sensation seeking, represented by a circle. Item errors/uniquenesses are indicated below each item. In addition, two error covariances are explicitly stated.

To investigate the structural validity (i.e., the robustness of the EFA structure), we conducted CFA for all eight items. We defined the single-factorial model as the baseline, in which all items were loaded on a single latent variable (Model 0). The baseline model showed an acceptable fit (Table 4). However, residual correlations, modification indices, and theoretical relevance, including item content, recommended the addition of error covariance between items 2 and 4. This modification resulted in Model 1, which performed significantly better than Model 0 ($\Delta \chi^{2}\left(1\right)=18.10$, p<0.001). In the final model, we also added covariance between the error terms of items 2 and 3 as suggested by the modification indices. This modification resulted in Model 2 performing significantly better than Model 1 ($\Delta \chi^{2}\left(1\right)=19.68$, p<0.001). The final model also demonstrated high factor loadings in the range of 0.34–0.90, and we assumed that the correlations between items 2–4 and 2–3 were associated with similar content. Figure 2 shows the structure of the final model, which includes the estimates of the standardized parameters. Although the results confirmed the one-factor model, we also considered a four-factor model as suggested by Zuckerman [2]. Unfortunately, the model-implied covariance matrix of the latent variables was not positive definite; therefore, we could not interpret the CFA.

### 3.3. Measurement Invariance

After the structural validity of the BSSS-8 was demonstrated, we investigated the measurement invariance using MG-CFA. Measurement invariance is necessary to confirm that the scale estimates the same construct for different groups. Table 5 summarizes the measurement invariance models for the BSSS-8. First, we tested for invariance across genders. The configural model proved to be well fitted and showed that the proposed MG-CFA model fit well with female and male participants. We also confirmed weak, strong, and strict invariance. Due to non-significant changes in model fit (ΔCFI<0.01), we confirmed the measurement invariance across genders.

Similarly, we tested for measurement invariance between the age groups. The participants were divided into two groups: young participants (≤20 years old, n=245) and older participants (>20 years old, n=118). The configural and weak models showed a good fit to the dataset. However, we could not confirm strong and strict invariance; differences in CFI were higher than the critical value (>0.01), reflecting model variance between age groups. We also tested for measurement invariance between education groups but could not confirm any degree of invariance (Table 5).

After confirming the gender-specific measurement invariance, we examined the differences between the genders, considering the BSSS-8 total score. The results showed a significant difference between male (M=23.90, SD=6.26) and female (M=22.00, SD=6.81) participants (t(102.10)=2.22, p=0.029), although the Cohen’s *d* was small (d=0.29).

Due to the unbalanced number of samples in the study groups, we repeated the analysis of measurement invariance using the Monte Carlo approach proposed by Yoon and Lai [61]. The subsampling procedure was repeated for the 100 simulated samples. Table 6 summarizes the simulation results. Consistent with the original data presented in Table 5, the simulation supported all three levels of invariance across genders only.

### 3.4. Nomological Network

The factorial validity described above is fundamental for a metrically adequate measuring instrument. However, does this also imply that the BSSS-8 scale really measures the trait it is supposed to capture [66]? To answer this question, we conducted zero-order correlation analysis to estimate the associations between the total BSSS-8 score and drug-related items.

**Figure 3:**
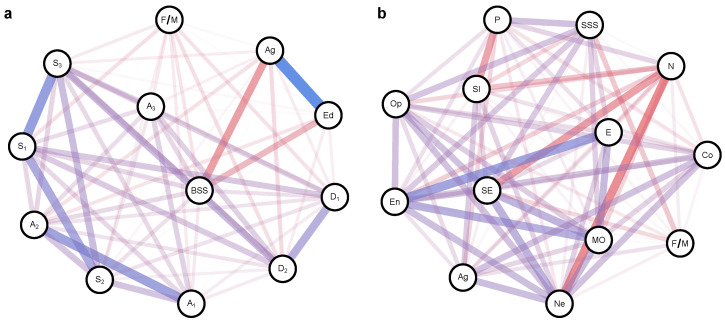
Nomological network describing the correlation structure between sensation seeking (SS) and related variables. Nodes refer to variables, whereas edges represent the correlations between variables. Positive correlations are depicted in blue, and negative correlations are shown in red. (**a**) The left panel represents the correlations between SS as measured by the Brief Sensation Seeking Scale (here abbreviated as BSS) and drug-related variables, including experience with alcohol (questions $A_{1}$–$A_{3}$), tobacco ($S_{1}$–$S_{3}$), illegal drugs ($D_{1}$, $D_{2}$), sex (F/M), age (Ag), and education level (Ed). (**b**) The right panel shows the correlations between SS as measured by the Sensation Seeking Scale-Form V (SSS-V); Eysenck personality traits, including Psychoticism (P), Extraversion (E), and Neuroticism (N); Big Five traits of Openness (Op), Conscientiousness (Co), Energy (En), Agreeableness (Ag), and Neuroticism (Ne); and dimensions reflecting the topology of the neural system as assessed by the Pavlov Temperament Survey, including Strength of Excitation (SE), Strength of Inhibition (SE), and Mobility (MO).

The left panel in Figure 3 shows the network representation of the correlation matrix between a set of variables. The nodes in the network refer to the measured variables, and the width of the edge represents the strength of the correlation between a pair of variables. The layout of the network was constructed using the algorithm proposed by [67], which places more influential and strongly connected nodes in the network closer together. As expected, the BSSS-8 score was significantly and positively correlated with all drug-related variables in the range 0.15–0.29. The total score was also negatively correlated with age (r=−0.53) and educational level (r=−0.41).

The right panel in Figure 3 shows the results of our earlier study on SS [38]. The correlations were based on a sample of 210 participants (139 females and 68 males) for whom complete data were available. SS was evaluated using the Zuckerman’s SSS-V scale. From the total item pool of 40 forced-choice items, we selected eight BSSS-8 items and computed the total score. As described in the Section 2, we also used (i) EPI, which measures personality traits according to the Eysenck PEN model; (ii) BFO, which evaluates prototypical personality traits defining each of the Big Five dimensions; and (iii) PTS, which provides psychological correlates of the primary CNS according to Pavlov’s understanding. As assumed, the SS construct correlated strongly with Extroversion, Psychoticism, Openness, Energy, and Strength of Excitation; the correlations ranged from 0.29 to 0.45. We also detected a negative correlation between SS and Strength of Inhibition (r=−0.24).

**Figure 4:**
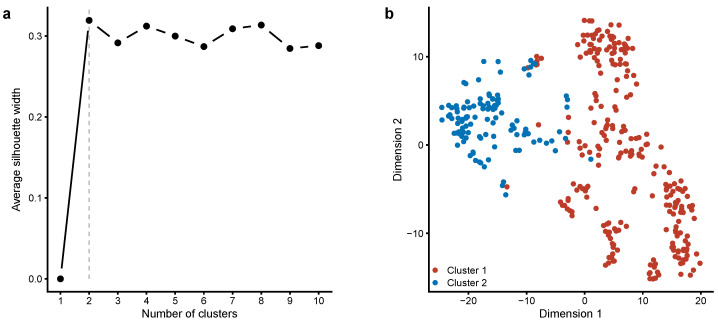
Clustering of participants based on Brief Sensation Seeking Scale scores, drug-related variables, and demographic data. (**a**) The partitioning around medoids algorithm is used to partition the dataset into two clusters, with the optimal number of clusters determined using the silhouette width method. (**b**) The multivariate dataset is represented using the t-distributed stochastic neighbor embedding (t-SNE) dimensionality reduction method. Both t-SNE axes are in arbitrary units. A detailed description of clusters is provided in the text.

To examine the inter-subject variability between the measured variables (i.e., BSSS-8 total score, drug-related variables, and demographic data), we used a partitioning around medoids clustering algorithm (a more robust version of the *k*-means algorithm) to find potential natural clusters in the data, followed by a t-SNE dimensionality reduction technique. Figure 4 shows all participants in an embedded space with two t-SNE dimensions. The plot shows two meaningful clusters: (i) Cluster 1 represents younger participants who scored high on the BSSS-8 and had high intentions regarding alcohol use and substance abuse, and (ii) Cluster 2 consists of older participants who scored low on the BSSS-8 and had lower intentions regarding alcohol use and substance abuse.

### 3.5. Item Response Theory Analysis

**Figure 5:**
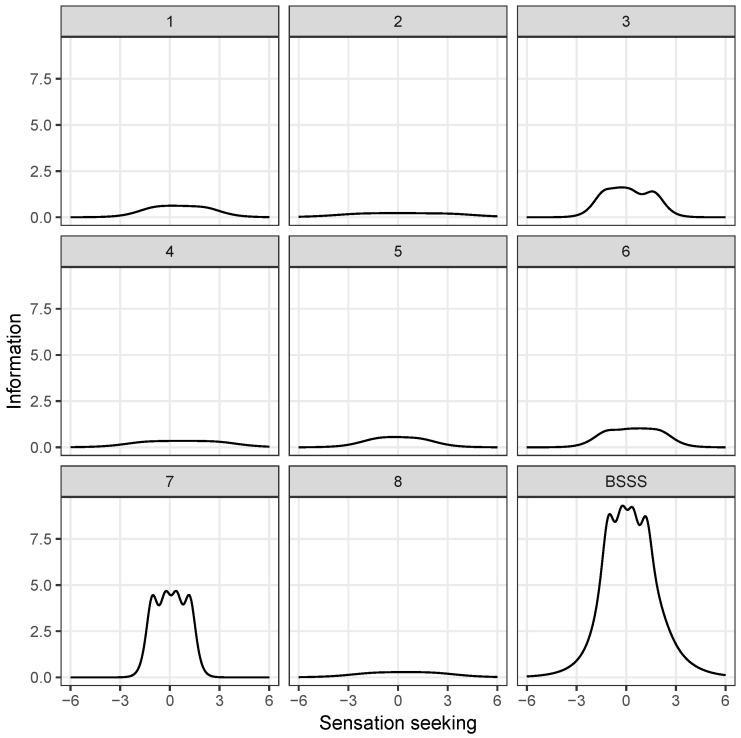
Item information function (IIF) curves (panels 1–8) and test information function (TIF) curve (panel BSSS). The IIF reflects the reliability of a particular item to estimate sensation seeking at different levels of the trait. The TIF represents the reliability of the entire Brief Sensation Seeking Scale. The TIF is the sum of the information provided by each item.

The results of the IRT analysis (Table 7) showed that the discrimination parameters were between 0.86 and 4.06, reflecting moderate to very high discrimination (i.e., the extent to which the item correlated with the latent trait) [68]. This indicates that the items were appropriately discriminated among the participants along the SS trait. Table 7 summarizes the estimated thresholds for all items. The thresholds ranged from −2.20 to 3.03. The thresholds in the fourth column ($\beta_{2}$) of Table 7 are similar to the mean value of the latent variable. For most items, a participant who had an average score for the latent trait of SS had approximately a 50% chance of either *disagreeing* (response value of 2) or *neither disagreeing nor agreeing* (response value of 3). As the slope and threshold parameters are often difficult to interpret in isolation, we utilized information functions that show the range of the latent variable over which each particular item is useful. Figure 5 shows the item information function (IIF) curves for all eight items and the test information function (TIF) curve. IIF represents the effectiveness of an item to measure the (personality) trait at different values of the trait continuum. On the other hand, the TIF shows the effectiveness of the entire scale [69]. The BSSS-8 items express information over a similar range of SS traits. The variation in the amount of information was small for all items. The items conveyed the same amount of information over the entire range of the latent variables. Items demonstrated the greatest informativeness, in the range of ±2 logits.

## 4. Discussion

Recently, the BSSS-8 was translated and adapted to various linguistic contexts. The main objective of this work was to provide a Slovenian version of the BSSS-8, accompanied by a comprehensive psychometric validation. The results showed that the Slovenian version of the BSSS-8 has good psychometric properties, including high reliability, good structural validity, and expected patterns of differences between genders and age groups. The CFA yielded a unidimensional factor structure. In addition, the single-factor solution proved to be invariant for both female and male participants. Nevertheless, it is imperative to acknowledge that further studies are required to determine the extent to which our findings can be generalized across genders, age cohorts, and educational levels.

A large amount of research has been conducted on sensation seeking SS over the last 40 years [4]. Various authors agree that SS is a psychological construct encompassing multiple dimensions. While the BSSS-8 was conceptualized based on Zuckerman’s four subfactors of SS, most researchers hypothesized that the scale primarily measures a single dimension of SS [28,33,34,70,71]. Our findings provide support for the unidimensional nature of the BSSS-8 and are in line with a comparable validation study of the BSSS in Spanish adolescents and young adults [35]. Considering the relatively small number of items, we believe that the unidimensional structure of BSSS-8 is more precise and parsimonious than the four-dimensional alternative. The results of the study also showed that the one-dimensional factor solution worked well for both male and female participants, whereas this was not the case with the four factors [72].

The translated version of the BSSS-8 showed good reliability, higher than that reported in previous studies [28,32]. In our study, the Cronbach’s α was above 0.80, which is the recommended value for acceptable reliability [73]. In addition to adequate reliability, the scale also showed good validity. Regarding construct validity, men scored significantly higher than women did. This finding is consistent with those of previous studies. For example, Zuckerman [3] reported that men scored higher than women on all subscales of the SSS-V, with the exception of the ES subscale. Cross et al. [74] conducted a comprehensive meta-analysis of gender differences in SS and showed that differences between females and males in SSS-V scores were stable over time, mainly due to the factors Dis and BS. In addition to gender, age was also significantly related to the BSSS-8 score in our study; younger participants reported significantly higher scores on SS than older participants. Zuckerman [2] postulated that SS increases in early childhood, peaks in adolescence, and decreases thereafter. Several authors have confirmed this assumption. For example, Steinberg et al. [75] and Khurana et al. [76] demonstrated that age differences in SS have a curvilinear shape, with a peak at approximately 15 years. In terms of concurrent validity, the BSSS-8 score was positively correlated with drug-related behaviors. This finding is consistent with the results of similar studies. A review of empirical evidence indicates that SS has strong predictive power for substance abuse and addiction [77]. Therefore, the BSSS-8 is a useful instrument for screening individuals whose high SS scores may correlate with (illegal) drug abuse.

Validation of the instrument for measurement invariance is an essential principle of modern psychometrics. We confirmed that the translated and adapted Slovenian version of the BSSS-8 is sex invariant. In less formal terms, this means that both women and men have similar conceptualizations of SS (configural invariance) and respond similarly to questionnaire items (weak invariance). In addition, it is possible to compare mean BSSS-8 total scores between sexes (strong invariance), and we can also compare raw BSSS-8 scores between women and men (strict invariance). In this study, measurement invariance across age groups and educational levels could not be confirmed. Comparisons of latent scores across age groups may be significantly biased, and differences in observed scores may not reflect true age-related differences in the SS. However, further studies with larger and more balanced groups (in terms of the desired grouping variables) are required.

We examined the intersubject variability of the included variables using a nonlinear multivariate analysis technique known as t-SNE. The t-SNE algorithm is a machine learning algorithm that facilitates the representation of a complex, high-dimensional dataset in a low-dimensional space. Using t-SNE, we were able to discriminate between the three homogeneous groups of participants. The algorithm clearly classified all “critical” participants with a high level of SS and a high probability of risky behavior into a single group. We attempted to replicate the t-SNE results using principal component analysis and multidimensional scaling but without success. In our opinion, t-SNE should receive more attention in current health research, including psychology.

This study provides further evidence for the validity of the BSSS-8 scale by comparing actual results with independent raw data from our previous study, which utilized the SSS-V scale in conjunction with two personality measures and a temperament survey. We selected eight BSSS items from a pool of 40 SSS-V items and computed the total score. The SS score based on the subset of SSS-V items showed the expected pattern of correlations with personality traits and temperament characteristics [7]. Specifically, our results demonstrated positive correlations between the SS score and Openness to Experience, Extroversion, and Psychoticism [78], providing additional support for the validity of the BSSS-8.

The major strengths of this study are the assessment of measurement invariance and the implementation of item response theory analysis. The translated scale will be particularly valuable in epidemiological research or as part of a larger battery of psychometric measures. To date, the Slovenian research community has used only outdated SSS-IV or inadequately adapted SSS-V scales. Last but not least, the translated scale will expand the existing psychological measurement tools used in research on factors contributing to risky behavior among Slovenian adolescents [79].

Along with the strengths described above, we acknowledge four limitations of the present study that should be considered in future research. First, and most importantly, a convenience sampling strategy resulted in a low proportion of males and a low proportion of older participants, which may have biased our conclusions. In the latter case, we discretized the age variable to distribute participants more evenly between the two groups. Furthermore, we repeated the standard procedure for measurement invariance using the Monte Carlo simulation to address the problem with imbalanced group sizes as proposed by Yoon and Lai [61] (see Section 2.4 and Section 3.3). The convenience sampling strategy is quick and cost effective but obviously results in sampling bias, limiting our ability to fully generalize the presented results. For example, a recent validation of the Dutch BSSS-8 scale reported an even higher discrepancy between the proportion of females and males [80]. Further research is required to thoroughly investigate the psychometric properties of the translated scale, using a more representative sample. Second, a longitudinal research design is required to estimate the metric characteristics of the scale over time. Validity would have been enhanced by comparing the BSSS-8 with other personality constructs (e.g., impulsiveness) using appropriate instruments. Third, future studies should systematically investigate the potential cultural biases that may arise in the linguistic adaptation process to ensure that the participants correctly understand the translated items. Finally, we did not control for the level of social desirability, although indicators of risk behaviors (even when a study is anonymous) may correlate with social desirability [81]. Despite these limitations, at the time of writing, the Slovenian version of the BSSS-8 is the most adequate instrument for assessing SS in Slovenia.

Knowledge of the SS construct is still incomplete. A thorough exploration of the biological mechanisms and societal factors underlying SS has not been conducted. We believe that functional brain imaging, molecular genetics, and modern statistical methods (e.g., complex network analysis) will provide further evidence for a better understanding of SS. We hope that our study will provide valuable insights in this direction.

## 5. Conclusions

In conclusion, this study successfully implemented the Slovenian version of the BSSS-8 and comprehensively validated its psychometric properties. The results revealed a unidimensional factor structure for the BSSS-8, robust internal consistency, and good concurrent validity. Building on the existing knowledge, we confirmed the previously reported positive correlation between SS and risk-taking behaviors.

The research community stands to benefit from the use of the translated BSSS-8 in situations where a simple, quick, and efficient assessment of SS is required, or in interdisciplinary studies involving a large number of questionnaires. We are optimistic that this study will stimulate and facilitate further research on SS in the Slovenian context.

## Figures and Tables

**Table 1:** Demographic characteristics of participants N=363.

Characteristic	*n* (%) ^1^
Gender	
Female	297 (82)
Male	66 (18)
Age (years)	
≤20	245 (68)
21–40	52 (14)
41–60	63 (17)
>60	3 (1)
Binarized age (years) ^2^	
≤20	245 (67)
>20	118 (33)
Education level	
Primary	103 (28)
Secondary	162 (45)
University or higher	98 (27)

^1^ Reported as no. (%) of participants. ^2^ Please see text for details.

**Table 2:** Descriptive statistics for the Brief Sensation Seeking Scale items.

Item	M (SD)	$\gamma_{1}$	$\gamma_{2}$	$r_{\mathrm{cor}}$	λ
(1) I like wild parties.	2.62 (1.31)	−0.24	−1.13	0.57	3.56
(2) I would like to explore strange places.	2.85 (1.23)	0.05	−1.11	0.44	1.02
(3) I like to do frightening things.	3.03 (1.21)	−0.24	−1.01	0.75	0.84
(4) I would like to take off on a trip with no pre-planned routes or timetables.	2.54 (1.21)	0.40	−0.84	0.54	0.70
(5) I would like to try bungee jumping.	3.10 (1.47)	−0.18	−1.41	0.53	0.61
(6) I prefer friends who are excitingly unpredictable.	2.57 (1.17)	0.40	−0.70	0.66	0.58
(7) I would love to have new and exciting experiences, even if they are illegal.	2.94 (1.27)	0.02	−1.07	0.84	0.44
(8) I get restless when I spend too much time at home.	2.71 (1.39)	0.30	−1.21	0.43	0.27

Note: $\gamma_{1}$ = skewness, $\gamma_{2}$ = kurtosis, $r_{\mathrm{cor}}$ = item whole correlation corrected for item overlap and scale reliability, λ = eigenvalue.

**Table 3:** Comparison of Brief Sensation Seeking Scale scores by gender, age, and education level.

	Gender			Age			Education			
	Male	Female		Young	Old		Primary	Secondary	University	
Item	M (SD)	M (SD)	*p*	M (SD)	M (SD)	*p*	M (SD)	M (SD)	M (SD)	*p*
1	2.97 (1.30)	2.54 (1.30)	0.049	2.97 (1.27)	1.90 (1.08)	0.001	2.96 (1.22)	2.88 (1.34)	1.84 (1.02)	0.001
2	3.17 (1.17)	2.78 (1.24)	0.049	2.85 (1.21)	2.86 (1.28)	0.961	3.03 (1.18)	2.70 (1.25)	2.91 (1.25)	0.250
3	3.33 (1.19)	2.97 (1.21)	0.053	3.33 (1.08)	2.41 (1.24)	0.001	3.20 (1.21)	2.35 (1.18)	2.35 (1.18)	0.001
4	2.53 (1.29)	2.55 (1.20)	0.931	2.69 (1.26)	2.25 (1.06)	0.001	2.54 (1.24)	2.30 (1.07)	2.30 (1.07)	0.015
5	3.06 (1.47)	3.11 (1.48)	0.930	3.50 (1.33)	2.26 (1.40)	0.001	3.37 (1.43)	2.21 (1.35)	2.21 (1.35)	0.001
6	2.74 (1.10)	2.53 (1.18)	0.259	2.82 (1.14)	2.05 (1.05)	0.001	2.72 (1.16)	2.72 (1.16)	1.89 (0.95)	0.001
7	3.38 (1.20)	2.85 (1.27)	0.013	3.31 (1.17)	2.17 (1.13)	0.001	3.27 (1.23)	3.27 (1.23)	2.04 (1.07)	0.001
8	2.76 (1.45)	2.70 (1.38)	0.930	2.87 (1.40)	2.38 (1.31)	0.001	2.96 (1.43)	2.96 (1.43)	2.21 (1.18)	0.050

Note: Raw *p*-values were adjusted for multiple testing.

**Table 4:** Goodness-of-fit indices for the proposed one-dimensional models.

Model	$\chi^{2}$	df	*p*	RMSEA	CFI	TLI
0	71.53	20	0.000	0.084 [0.065, 0.105]	0.932	0.904
1	50.49	19	0.000	0.068 [0.046, 0.089]	0.958	0.938
2	30.48	18	0.033	0.044 [0.014, 0.069]	0.983	0.974

Note: $\chi^{2}$ = model $\chi^{2}$ statistic, *df* = degrees of freedom, *p* = *p*-value, RMSEA = Root Mean Square Error of Approximation, CFI = Comparative Fit Index, TLI = Tucker-Lewis Index (TLI). Model 0 = baseline unidimensional model, Model 1 = baseline model including covariances between items 2 and 4, Model 2 = baseline model including covariances between items 2 and 4, and 2 and 3.

**Table 5:** Goodness-of-fit indices for measurement invariance by gender, age, and education level.

Model	$\chi^{2}$	df	*p*	RMSEA	CFI	ΔCFI
Gender						
Configural	45.06	36	0.143	0.039	0.988	/
Weak	53.46	44	0.155	0.035	0.988	0.000
Strong	67.86	51	0.057	0.044	0.978	−0.009
Strict	79.33	59	0.040	0.044	0.976	−0.003
Age						
Configural	49.38	36	0.068	0.047	0.979	/
Weak	62.53	44	0.034	0.050	0.971	−0.008
Strong	96.42	51	0.001	0.073	0.929	−0.042
Strict	117.43	59	0.001	0.076	0.910	−0.019
Education						
Configural	66.62	54	0.116	0.045	0.981	/
Weak	92.18	70	0.039	0.052	0.966	−0.015
Strong	134.69	84	0.001	0.073	0.922	−0.044
Strict	170.94	100	0.001	0.078	0.894	−0.028

Note: $\chi^{2}$ = model $\chi^{2}$ statistic, *df* = degrees of freedom, *p* = *p*-value, RMSEA = Root Mean Square Error of Approximation, CFI = Comparative Fit Index, TLI = Tucker–Lewis Index (TLI). Cut-off value for measurement invariance is ΔCFI≤0.01 [60]. Males (n=66), females (n=297), young (n=245), older (n=118); primary school (n=103), secondary school (n=162), university (n=98).

**Table 6:** Goodness-of-fit indices for the forced-balanced dataset using a subsampling approach.

Model	$\chi^{2}$	RMSEA	CFI	ΔCFI
Gender				
Configural	38.10 (8.48)	0.030 (0.031)	0.985 (0.022)	/
Weak	45.33 (8.47)	0.023 (0.028)	0.987 (0.021)	−0.002
Strong	56.71 (9.33)	0.036 (0.028)	0.977 (0.027)	0.010
Strict	64.00 (9.93)	0.030 (0.028)	0.978 (0.028)	−0.001
Age				
Configural	47.48 (7.50)	0.049 (0.019)	0.976 (0.015)	/
Weak	59.58 (8.58)	0.052 (0.017)	0.969 (0.017)	−0.007
Strong	88.99 (9.48)	0.079 (0.010)	0.923 (0.018)	−0.046
Strict	105.65 (10.06)	0.081 (0.009)	0.906 (0.021)	−0.017
Education				
Configural	69.02 (6.61)	0.052 (0.012)	0.974 (0.011)	/
Weak	94.64 (7.89)	0.059 (0.010)	0.957 (0.013)	−0.002
Strong	134.68 (9.86)	0.078 (0.008)	0.912 (0.016)	−0.003
Strict	163.60 (10.22)	0.080 (0.006)	0.889 (0.017)	−0.001

Note: The entries in the table are the Monte Carlo averages of the indices for 100 generated samples. The corresponding standard deviations are given in parentheses. $\chi^{2}$ = model $\chi^{2}$ statistic, RMSEA = Root Mean Square Error of Approximation, CFI = Comparative Fit Index. Cut-off values for measurement invariance are ΔCFI≤0.01 [60]. Males (n=66), females (n=297), young (n=245), older (n=118); primary school (n=103), secondary school (n=162), university (n=98).

**Table 7:** Discrimination and threshold parameters for Brief Sensation Seeking Scale items.

Item	α	$\beta_{1}$	$\beta_{2}$	$\beta_{3}$	$\beta_{4}$
1	1.41	−1.31	−0.09	1.18	2.92
2	0.86	−1.88	−0.29	0.65	2.58
3	2.33	−3.09	−1.09	0.58	3.81
4	1.06	−1.48	0.18	1.37	2.97
5	1.33	−1.63	−0.60	0.07	1.66
6	1.85	−2.14	0.15	1.86	3.71
7	4.06	−4.31	−1.06	1.64	4.84
8	0.95	−1.35	0.03	0.84	2.01

Note: α = discrimination parameter, $\beta_{1}$–$\beta_{4}$ = threshold parameter.

## Data Availability

The data presented in this study are available on request from the corresponding author.

## Abbreviations

The following abbreviations are used in this manuscript:

AISS	Arnett Inventory of Sensation Seeking
BFO	Big Five Observer
BS	Boredom Susceptibility
BSSS-8	Brief Sensation Seeking Scale
CFA	Confirmatory Factor Analysis
CFI	Comparative Fit Index
CNS	Central Nervous System
EFA	Exploratory Factor Analysis
EPQ	Eysenck Personality Questionnaire
EPQR-S	Eysenck Personality Questionnaire-Revised Short Form
ES	Experience Seeking
Dis	Disinhibition
GRM	Graded Response Model
IFF	Item Information Function
IRT	Item Response Theory
KMO	Kaiser–Meyer–Olkin index
MG-CFA	Multi-Group Confirmatory Factor Analysis
MO	Mobility
PTS	Pavlovian Temperament Survey
RMSEA	Root Mean Square Error of Approximation
SE	Strength of Excitation
SI	Strength of Inhibition
SS	Sensation Seeking
SSS	Sensation Seeking Scale
SSS-IV	Sensation Seeking Scale-Form IV
SSS-V	Sensation Seeking Scale-Form V
TAS	Thrill and Adventure Seeking
TIF	Test Information Function
TLI	Tucker–Lewis index
t-SNE	t-distributed Stochastic Neighbor Embedding
ZKA-PQ	Zuckerman–Kuhlman–Aluja Personality Questionnaire

## Appendix A

**Table A1:** Slovenian version of Brief Sensation Seeking Scale.

Item	Subscale	Item Question
1	Dis	I like wild parties. (Rad imam divje zabave brez zadržkov.)
2	ES	I would like to explore strange places. (Všeč mi je, da sam raziskujem mesto, pa čeprav se pri tem izgubim.)
3	TAS	I like to do frightening things. (Včasih rad počnem stravi, ki so malo zastrašujoče.)
4	ES	I would like to take off on a trip with no pre-planned routes or timetables. (Rad potujem brez vsakih vnaprejšnjih načrtov, ciljev ali urnikov.)
5	TAS	I would like to try bungee jumping. (Rad bi se naučil skakati s padalom.)
6	BS	I prefer friends who are excitingly unpredictable. (Rad imam prijatelje, pri katerih je najbolj vznemirljivo to, da nikoli ne veš, kaj bodo storili.)
7	Dis	I would love to have new and exciting experiences, even if they are illegal. (Všeč so mi nova in razburljiva doživetja, tudi če so nenavadna, zastrašujoča ali prepovedana.)
8	BS	I get restless when I spend too much time at home. (Če moram dlje časa ostati doma, hitro postanem nemiren.)

Note: TAS = Thrill and Adventure Seeking, ES = Experience Seeking, Dis = Disinhibition, BS = Boredom Susceptibility. The Slovenian translation is provided in parentheses.

**Table A2:** Comparison of Brief Sensation Seeking Scale scores by gender, age, and education level.

	Gender					Age					Education					
	Male	Female	Difference			Young	Old	Difference			Primary	Secondary	University	Difference		
Item	M (SD)	M (SD)	t	*p*	d	M (SD)	M (SD)	t	*p*	d	M (SD)	M (SD)	M (SD)	F	*p*	$\eta^{2}$
1	2.97 (1.30)	2.54 (1.30)	2.41	0.049	0.33	2.97 (1.27)	1.90 (1.08)	8.32	0.001	0.88	2.96 (1.22)	2.88 (1.34)	1.84 (1.02)	28.25	0.001	0.035
2	3.17 (1.17)	2.78 (1.24)	2.39	0.049	0.31	2.85 (1.21)	2.86 (1.28)	−0.05	0.961	−0.01	3.03 (1.18)	2.70 (1.25)	2.91 (1.25)	1.33	0.250	0.002
3	3.33 (1.19)	2.97 (1.21)	2.25	0.053	0.30	3.33 (1.08)	2.41 (1.24)	6.97	0.001	0.82	3.20 (1.21)	2.35 (1.18)	2.35 (1.18)	34.14	0.001	0.034
4	2.53 (1.29)	2.55 (1.20)	−0.09	0.931	−0.01	2.69 (1.26)	2.25 (1.06)	3.48	0.001	0.37	2.54 (1.24)	2.30 (1.07)	2.30 (1.07)	8.00	0.015	0.011
5	3.06 (1.47)	3.11 (1.48)	−0.24	0.930	−0.03	3.50 (1.33)	2.26 (1.40)	8.01	0.001	0.91	3.37 (1.43)	2.21 (1.35)	2.21 (1.35)	30.96	0.001	0.036
6	2.74 (1.10)	2.53 (1.18)	1.41	0.259	−0.18	2.82 (1.14)	2.05 (1.05)	6.31	0.001	0.69	2.72 (1.16)	2.72 (1.16)	1.89 (0.95)	37.78	0.001	0.041
7	3.38 (1.20)	2.85 (1.27)	3.23	0.013	0.42	3.31 (1.17)	2.17 (1.13)	8.96	0.001	0.99	3.27 (1.23)	3.27 (1.23)	2.04 (1.07)	35.47	0.001	0.037
8	2.76 (1.45)	2.70 (1.38)	0.28	0.930	0.04	2.87 (1.40)	2.38 (1.31)	3.28	0.001	0.36	2.96 (1.43)	2.96 (1.43)	2.21 (1.18)	5.08	0.050	0.008

Note: Raw *p*-values were adjusted for multiple testing. Effect sizes for all variables were expressed as Cohen’s *d* or $\eta^{2}$ as appropriate. For the interpretation of effect sizes, please see the text.
